# Effects of EGFR Inhibitor on *Helicobacter pylori* Induced Gastric Epithelial Pathology *in Vivo*

**DOI:** 10.3390/pathogens2040571

**Published:** 2013-10-14

**Authors:** Jean E. Crabtree, Anthony H.T. Jeremy, Cedric Duval, Michael F. Dixon, Kazuma Danjo, Ian M. Carr, D. Mark Pritchard, Philip A. Robinson

**Affiliations:** 1 Leeds Institute of Molecular Medicine, St. James’s University Hospital, Leeds LS9 7TF, UK; E-Mails: Anthony.jeremy@evocutis.com (A.H.T.J.); C.Duval@leeds.ac.uk (C.D.); kaz.danjo@nifty.com (K.D.); I.M.Carr@leeds.ac.uk (I.M.C.); 2 Department of Pathology, University of Leeds, Leeds LS2 9JT, UK; E-Mail: mikedixon@blueyonder.co.uk; 3 Department of Gastroenterology, Institute of Translational Medicine, University of Liverpool, Liverpool L69 3GA, UK; E-Mail: Mark.Pritchard@liverpool.ac.uk

**Keywords:** *Helicobacter pylori*, EGFR inhibitor, EKB-569, epithelial cell proliferation, apoptosis

## Abstract

*Helicobacter pylori* transactivates the Epidermal Growth Factor Receptor (EGFR) and predisposes to gastric cancer development in humans and animal models. To examine the importance of EGFR signalling to gastric pathology, this study investigated whether treatment of Mongolian gerbils with a selective EGFR tyrosine kinase inhibitor, EKB-569, altered gastric pathology in chronic *H. pylori* infection. Gerbils were infected with *H. pylori* and six weeks later received either EKB-569-supplemented, or control diet, for 32 weeks prior to sacrifice. EKB-569-treated *H. pylori-*infected gerbils had no difference in *H. pylori* colonisation or inflammation scores compared to infected animals on control diet, but showed significantly less corpus atrophy, mucous metaplasia and submucosal glandular herniations along with markedly reduced antral and corpus epithelial proliferation to apoptosis ratios. EKB-569-treated infected gerbils had significantly decreased abundance of *Cox-2*, *Adam17* and *Egfr* gastric transcripts relative to infected animals on control diet. EGFR inhibition by EKB-569 therefore reduced the severity of pre-neoplastic gastric pathology in chronically *H. pylori-*infected gerbils. EKB-569 increased gastric epithelial apoptosis in *H. pylori-*infected gerbils which counteracted some of the consequences of increased gastric epithelial cell proliferation. Similar chemopreventative strategies may be useful in humans who are at high risk of developing *H. pylori-* induced gastric adenocarcinoma.

## 1. Introduction

Infection with the gastric bacterium *Helicobacter pylori* is associated with increased risk of developing distal gastric cancer [1,2]. The risk of gastric cancer is greatest in those with non-ulcer dyspepsia, or gastric ulceration, who develop gastric atrophy and intestinal metaplasia with long term *H. pylori* infection [3]. The chronic inflammatory response to *H. pylori* [1,2] and pathogen stimulated epithelial signalling responses [4,5]) contribute to an increased risk of neoplasia. *H. pylori* strains with the *cag* pathogenicity island (*cag* PAI), a type IV secretory system [6], are associated with both increased inflammation and epithelial cell signalling responses [1,2,4,5].

Marked gastric epithelial hyperplasia with *H. pylori* infection occurs in both humans and rodent models [7,8,9,10,11,12]. EGFR transactivation has been strongly implicated in epithelial hyperproliferation and cancer [13]. Early studies identified that both *cag* PAI positive and negative *H. pylori* strains transactivate the EGFR on gastric epithelial cells [14,15,16] similarly to bacterial pathogens infecting other sites such as *Pseudomonas aeruginosa* [17]. *H. pylori*-stimulated EGFR transactivation is dependent on stimulation of heparin binding-epidermal growth factor (HB-EGF) which requires metalloprotease, EGFR and Mek-1 activities [14]. *H. pylori* induced cleavage of membrane bound proHB-EGF is mediated by a disintegrin and matrix metalloprotease-17 (ADAM17) [18]. Both gastric ADAM17 [19], and EGFR ligands HB-EGF, amphiregulin and EGF [20,21,22], are increased in patients with *H. pylori* infection and/or gastric cancer and are likely to contribute to epithelial hyperplasia. Additionally *H. pylori* upregulates EGFR in cultured gastric epithelial cells [23] and blocks EGFR endocytosis [24].

Whilst many studies have investigated *H. pylori* stimulated EGFR signalling responses *in vitro* [4,5,14,15,16,25,26], the importance of *H. pylori* EGFR transactivation *in vivo* in chronic infection has not been investigated. Mongolian gerbils have been extensively used for investigating *H. pylori-*induced gastritis [9,10,27] which can result in gastric cancer with longer term infection [28,29]. *H. pylori* infection in gerbils, in contrast to mice, induces severe antral active chronic gastritis, which progresses to pan gastritis with corpus atrophy [9,10]. Infection is associated with epithelial hyperproliferation [9,10] and with long term infection apoptosis in epithelial cells, which is initially increased, decreases [10]. As EGFR hyperactivity is considered critical in the initiation and progression of epithelial derived tumours, EGFR tyrosine kinase inhibitors have therapeutic potential as chemopreventative agents for gastrointestinal neoplasia [30]. Whilst the chemotherapeutic effects of EGFR-kinase inhibitors on intestinal neoplasia is established in mice [30], their potential for preventing *H. pylori-*induced hyperproliferative disease is unknown. 

The aims of this study were to investigate the effects of a selective EGFR tyrosine kinase inhibitor, EKB-569 [30] on *H. pylori-*induced gastric pathology, epithelial hyperproliferative responses and expression of genes in the EGFR triple membrane passing signalling (TMPS) cascade which are essential for EGFR transactivation [31]. Our previous longitudinal studies on pathology, epithelial proliferation and apoptosis in *H. pylori* SS1 strain infected Mongolian gerbils identified a significant progression to corpus atrophy and reduction in gastric epithelial cell apoptosis at 36 weeks post-infection [10]. Corpus atrophy is a recognised pre-cursor condition for gastric cancer [1,2,3]. Based on these earlier kinetic studies [10], the effects of EKB-569 treatment in *H. pylori* SS1 strain infected Mongolian gerbils were examined at 38 weeks post-infection to assess the effect of treatment on the development of the pre-neoplastic lesion corpus atrophy and associated changes in epithelial cell apoptosis, proliferation and gene expression.

## 2. Results

### 2.1. EKB-569 Effects on H. pylori-Induced Epithelial Responses *in Vitro*

Previous *in vitro* studies using “In Cell Western” analysis identified that EKB-569 significantly inhibited *H. pylori*-stimulated ERK1/2 phosphorylation (pERK1/2) in A-431 cells [16]. Inhibitory effects were not specific to strains with a functional *cag* PAI*.* ERK1/2 phosphorylation in A-431 cells was examined as a readout of EGFR signalling rather than phosphorylated EGFR due to cross reactivity of pEGFR antibodies with *H. pylori* [16]. Initial *in vitro* studies determined whether EKB-569 inhibited SS1-induced pERK1/2 in A431 cells. At 100nm and 1,000nm EKB-569 inhibited *circa* 70% SS1-induced pERK1/2 relative to untreated SS1-stimulated cells (Table 1).

**Table 1:** Inhibition of ERK1/2 phosphorylation induced by *H. pylori* SS1 strain in A-431 epithelial cells by EKB-569. A-431 cells were co-incubated with *H. pylori* strain SS1 for 180 min with, or without, 60 min EKB-569 pre-incubation. Unstimulated A-431 cells were similarly pre-incubated with, and without, EKB-569. ERK1/2 phosphorylation in *H. pylori* stimulated and unstimulated cells was quantified by “In Cell Western” analysis of the 700/800 nm Relative Response (RR) [16]. Values represent net values after subtraction of the effect of EKB-569 on the endogenous ERK1/2 phosphorylation in unstimulated cells. The significance between control *H. pylori* SS1 and EKB-569 treated groups was analysed by paired *t*-test (* *p* < 0.05; ** *p* < 0.001, Values are mean ± SEM of 4 independent experiments.

EKB-569 nM	0	1 nM	10 nM	100 nM	1,000 nM
700/800 nm RR	65 ± 3 (100%)	31 ± 10 * (48%)	21 ± 7 ** (33%)	19 ± 8 ** (29%)	20 ± 5 ** (32%)

### 2.2. H. pylori Infection of Mongolian Gerbils

The effects of EKB-569 treatment on gastric pathology and corpus atrophy were examined at 38 weeks post-infection. At 38 weeks post-inoculation all gerbils on control diet (n = 7) or EKB-569 diet (n = 8) were *H. pylori* positive by microbial culture. Additionally, all inoculated gerbils on EKB-569 and control diet were classified histologically as *H. pylori* infected. No control gerbils were positive for *H. pylori* by histology or microbial culture (n = 10 per group). There was no significant difference in *H. pylori* antral density in EKB-569 treated gerbils (density grade mean ± SEM = 1.38 ± 0.20) compared to gerbils on control diet (1.43 ± 0.26), or in the corpus mucosa (control and EKB-569 diet, 1.0 ± 0.0). 

### 2.3. Gastric Pathology in EKB-569 Treated and Control H. pylori-Infected Gerbils

**Figure 1:**
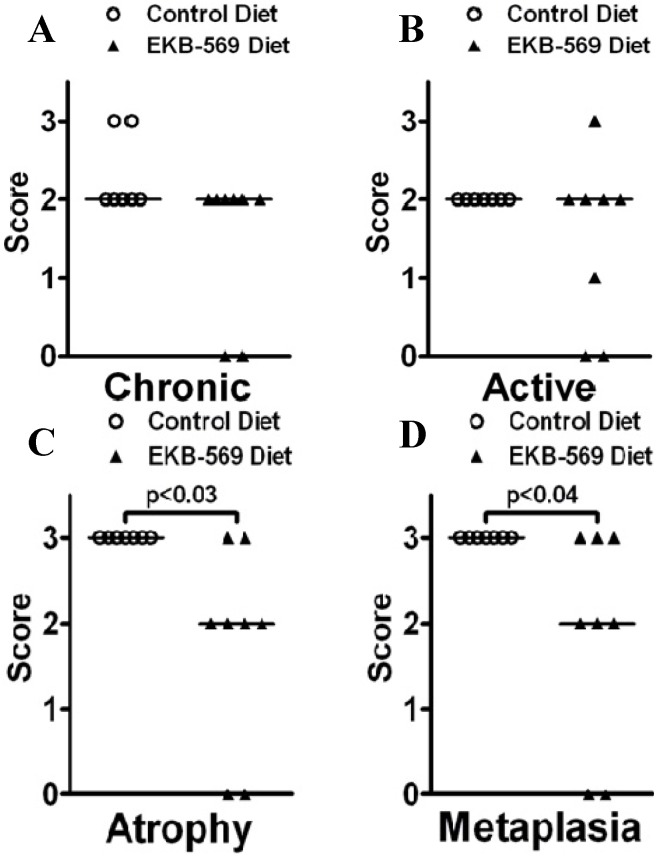
Gastric corpus histopathology scores in *H. pylori* infected Mongolian gerbils fed Epidermal Growth Factor Receptor (EGFR) inhibitor diet or control diet; (**A**) chronic inflammation scores; (**B**) active inflammation scores; (**C**) corpus atrophy scores; (**D**) mucous metaplasia scores. Open circles gerbils on control diet, closed triangles gerbils on EKB-569 diet, n = 7–8 per group. Comparisons between groups were made using stratified Chi squared for trend. Bars represent median scores.

In *H. pylori-*infected gerbils, chronic and active inflammation and atrophy were evident in the antrum at 38 weeks post-infection, but there was no significant difference in scores between infected gerbils on control diet and EKB-569 diets (data not shown). In the corpus mucosa, chronic and active inflammatory scores in *H. pylori-*infected gerbils did not differ between EKB-569 treated and untreated gerbils (Figure 1A,B). In contrast, corpus atrophy (Figure 1C) and corpus mucous metaplasia (Figure 1D) in *H. pylori-*infected gerbils was significantly lower (*p* < 0.05) in EKB-569 treated gerbils. Uninfected control gerbils on normal diet and EKB-569 diet had histologically normal antral and corpus mucosa. 

**Figure 2:**
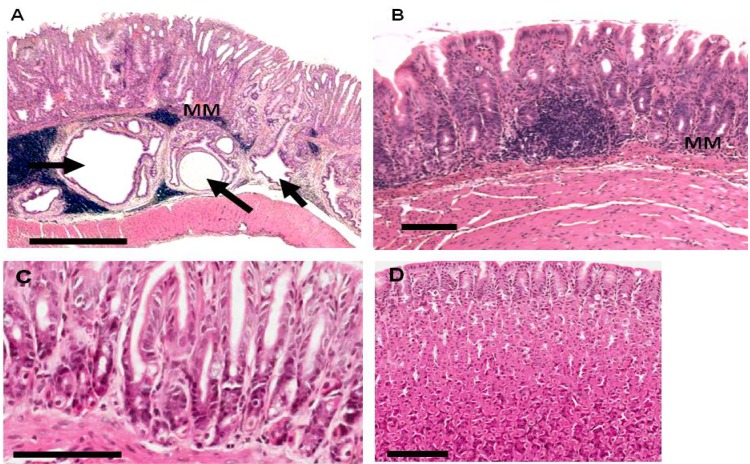
Pathology of gastric mucosa of *H. pylori* infected Mongolian gerbils and uninfected gerbils fed EGFR inhibitor diet or control diet. Haematoxylin and eosin stained sections of gastric antral mucosa of Mongolian gerbils 38 weeks post-infection with *H. pylori* SS1 strain. (**A**) *H. pylori* infected gerbil on control diet, Bar 800 µm; MM-muscularismucosa which is discontinuous on the right hand side, three black arrows indicate sub-mucosal glandular herniations. The right hand sub-mucosal herniation is sectioned longitudinally reaching the gastric lumen; (**B**) Antral mucosa of infected gerbil on EKB-569 diet lacking sub-mucosal herniations, Bar-100 μm; (**C**) Antral mucosa of uninfected gerbil on control diet, Bar-100 µm; (**D**) Corpus mucosa of uninfected gerbil treated with EKB-569, Bar-100 μm.

At 38 weeks six of seven (86%) *H. pylori-*infected gerbils on control diet had frequent submucosal glandular herniations (Figure 2A). EKB-569 treatment resulted in a significant reduction (*p* < 0.05, Fisher’s exact test) in glandular herniations which were present in two of eight (25%) of *H. pylori-*infected EKB-569 treated gerbils (Figure 2B). Herniation scores were significantly higher (*p* < 0.05, Chi-squared test for trend) in infected gerbils on control diet (1.57 ± 0.37) than EKB-569 treated gerbils (0.50 ± 0.38). Uninfected control gerbils on normal diet and EKB-569 diet had no herniations (Figure 2C,D).

### 2.4. EGFR Inhibitor Reduces Phosphorylated Erk Positive Gastric Epithelial Cells in H. pylori-Infected Gerbils

**Figure 3:**
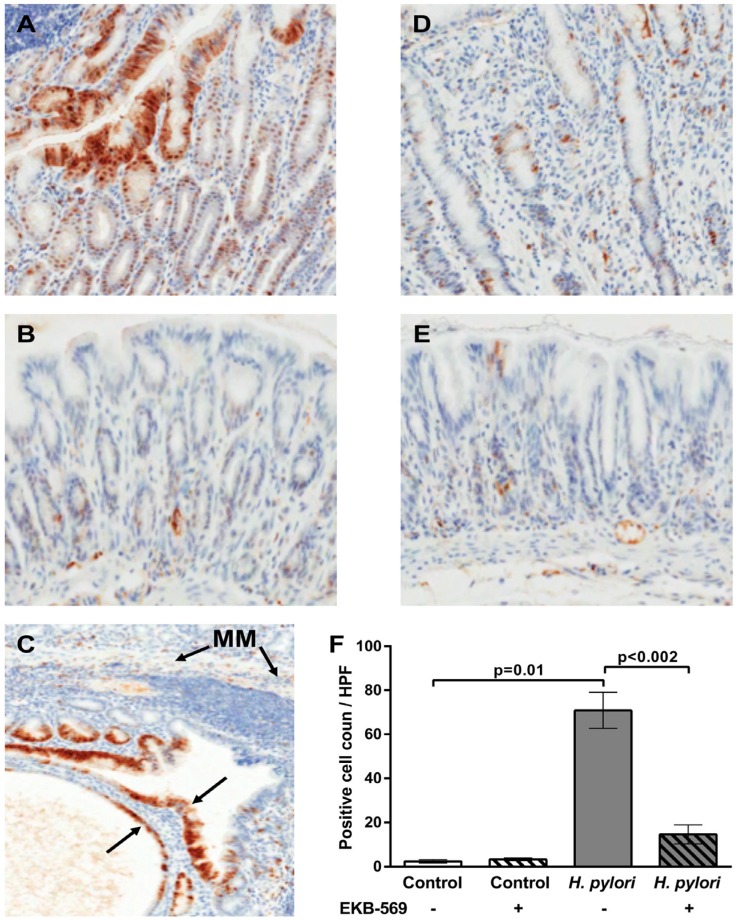
Immunohistological analysis of phospho-Erk labelling in the gastric mucosa of *H. pylori* SS1 strain infected, and uninfected, Mongolian gerbils treated with EGFR inhibitor diet or control diet. The brown precipitate is diaminobenzidine staining indicating phospho-Erk 1/2. Sections have been counterstained with haematoxylin. Representative images from sections of gastric antral mucosa of Mongolian gerbils 38 weeks post-infection. (**A**) *H. pylori* infected gerbil on control diet showing phospho-Erk positive gastric epithelial cells and lamina propria cells. Magnification ×130; (**B**) Antral mucosa of uninfected gerbil on control diet. Magnification ×140; **(C)** Antral mucosa of *H. pylori* infected gerbil on control diet showing two large submucosal herniations with phospho-Erk positive epithelial cells (black arrows). Magnification, ×120. MM-muscularis mucosa; (**D**) Antral mucosa of *H. pylori* infected gerbil treated with EKB-569 diet showing reduced phospho-Erk labelling in gastric epithelial cells. Magnification ×140; (**E**) Antral mucosa of uninfected gerbil on EKB-569 diet. Magnification ×140; (**F**) Number of phospho-Erk positive gastric epithelial cells per high-powered field (n = 5) in *H. pylori* infected (n = 7 control diet; n = 8 EKB-569 diet) and uninfected Mongolian gerbils (n = 4 control diet; n = 4 EKB-569 diet). Data are expressed as mean ± SEM. Statistical analysis Mann Whitney U test.

Immunohistochemistry was used to assess phosphorylation of Erk1/2 in the gastric mucosa in the Mongolian gerbils. At 38 weeks post-infection *H. pylori* infected gerbils on control diet had significantly increased (*p* = 0.01) phosphorylated Erk1/2 gastric epithelial cells relative to the uninfected controls (Figure 3A,B). Both cytoplasmic, and in some instances, nuclear phosphorylated Erk positive epithelial cells were evident mainly in the upper parts of the gastric mucosa in *H. pylori* infected gerbils on the control diet (Figure 3A). However, phospho-Erk positivity was also evident in gastric epithelial cells in the sub-mucosal glandular herniations in infected gerbils on control diet (Figure 3C). Treatment of *H. pylori* infected gerbils with EKB-569 resulted in a significant reduction in phospho-ERK positive epithelial cells compared to *H. pylori* infected gerbils on control diet (Figure 3D). Phospho-Erk1/2 positive mononuclear cells in the lamina propria were evident in *H. pylori* infected gerbils both on the control diet and EKB-569 diet. In uninfected gerbils on control diet and EKB-569 diet there were very few phospho-Erk positive gastric epithelial or lamina propria cells (Figure 3B,E,F). The number of phospho-Erk positive gastric epithelial cells per high-power field (×40) in uninfected and *H. pylori* infected gerbils on control and EKB-569 diet is shown in Figure 3F. The results indicate that EKB-569 significantly reduces *H. pylori* induced gastric phospho-Erk signalling in gastric epithelial cells *in vivo* (*p* < 0.002) (Figure 3F) as well as *in vitro* (Table 1).

### 2.5. EGFR Inhibitor Modifies Gastric Epithelial Cell Proliferation Apoptosis Ratios in H. pylori- Infected Gerbils

**Figure 4:**
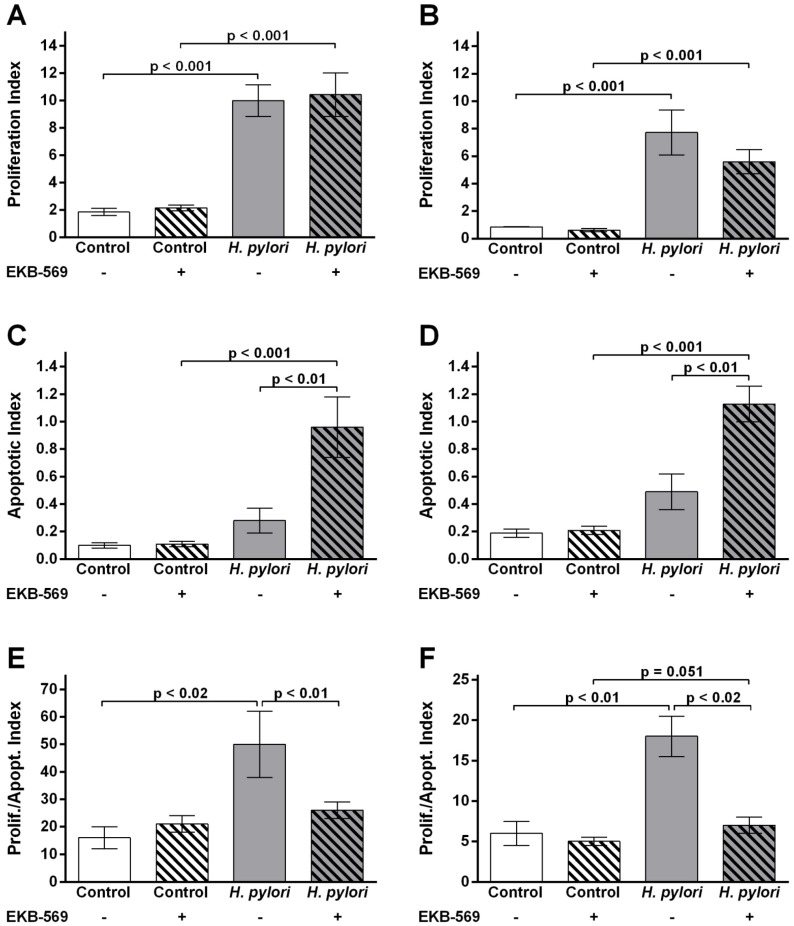
EGFR inhibitor increases apoptosis and decreases gastric epithelial proliferation to apoptosis ratio in *H. pylori* infected gerbils. (**A**) Epithelial cell proliferation labelling index (LI%) in the antrum of Mongolian gerbils infected with *H. pylori* SS1 strain and uninfected controls fed EKB-569 containing diet or control diet; (**B**) Epithelial cell proliferation labelling index (LI%) in the corpus of Mongolian gerbils infected with *H. pylori* SS1 strain and uninfected controls fed EKB-569 containing diet or control diet; (**C**) Epithelial cell apoptosis index (AI%) in the glandular epithelium in the antrum of Mongolian gerbils infected with *H. pylori* SS1 strain and uninfected controls fed EKB-569 containing diet or control diet; (**D**) Epithelial cell apoptosis index (AI%) in the glandular epithelium in the corpus of Mongolian gerbils infected with *H. pylori* SS1 strain and uninfected controls fed EKB-569 containing diet or control diet; (**E**) Proliferation/apoptosis ratio in antrum in infected and control gerbils fed EKB-569 containing diet or control diet; (**F**) Proliferation/apoptosis ratio in corpus of infected and control gerbils fed EKB-569 containing diet or control diet. Group size n = 7–10 gerbils. Grey histograms *H. pylori* infected gerbils on EKB-569 diet and control diet; white bars uninfected gerbils on EKB-569diet and control diet. Hatched bars infected and control groups treated with EKB-569 diet; unhatched bars infected and control groups on control diet. Data are expressed as mean ± SEM. Statistical analysis Mann-Whitney U test.

In uninfected animals no differences in epithelial proliferation or apoptosis were observed between gerbils on control or EKB-569 diet (Figure 4A–D). At 38 weeks post-infection there was a significant increase in antral (*p* < 0.001) and corpus (*p* < 0.001) epithelial cell proliferation in EKB-569 treated and untreated gerbils compared to uninfected controls (Figure 4A–B). In infected gerbils the corpus epithelial proliferation index in EKB-569 treated gerbils was lower than that of infected gerbils on control diet (Figure 4B). Antral and corpus epithelial apoptosis was significantly increased in *H. pylori-*infected gerbils treated with EKB-569 compared to *H. pylori* positive gerbils on control diet (*p* < 0.01) and uninfected control groups (*p* < 0.001) (Figure 4C,D). 

The ratio of gastric epithelial cell proliferation to apoptosis in EKB-569 treated control and *H. pylori* infected gerbils is shown in Figure 3E,F. In uninfected gerbils, EKB-569 treatment had no effect on the proliferation to apoptosis ratio. In *H. pylori* infected gerbils on control diet there was a significant increase in antral epithelial cell proliferation to apoptosis ratio compared to uninfected controls (*p* < 0.02) and the *H. pylori* infected group treated with EKB-569 (*p* < 0.01). EKB-569 treated infected gerbils had an antral proliferation to apoptosis ratio comparable to uninfected control groups (Figure 4E). In the corpus the gastric epithelial proliferation to apoptosis ratio was similarly significantly increased in *H. pylori* infected gerbils on control diet relative to uninfected controls (*p* < 0.01) and the *H. pylori* infected group treated with EKB-569 (*p* < 0.02) (Figure 4F). The EKB-569 treated infected gerbils had a corpus proliferation to apoptosis ratio comparable to uninfected untreated controls, but was greater than the uninfected EKB-569 treated group (*p* = 0.05).

### 2.6. Sequence Analysis of Gerbil Transcripts

To facilitate analysis of the effects of EKB-569 on gastric expression of genes involved in *H. pylori* induced EGFR transactivation, RT-PCR products produced from transcripts coding for *Adam17*, *Egfr* and *Hb-Egf* were analysed by agarose gel electrophoresis, DNA bands were excised and gerbil DNA sequenced. The sequences were aligned with respective rodent and human sequences and showed a high degree of homology (Figure S1). The 213 base pair nucleotide encoding for Mongolian gerbil *Adam17* (EMBL Accession number HF679116) was 95%, 95%, 95% and 94% identical to mouse, rat, Chinese hamster and human orthologues, respectively (Figure S1A). The 216 base pair nucleotide encoding Mongolian gerbil *Egfr* (EMBL Accession number HF679117) was 92%, 91% and 80% identical to mouse, rat and human orthologues respectively (Figure S1B). The 200 base pair nucleotide encoding Mongolian gerbil *Hb-egf* (EMBL Accession number HF679118) was 93%, 93%, 90% and 79% identical to mouse, rat, Chinese hamsters and human orthologues, respectively (Figure S1C). 

### 2.7.Effects of EGFR Inhibitor on H. pylori Induced Gastric Gene Expression in Gerbils

**Figure 5:**
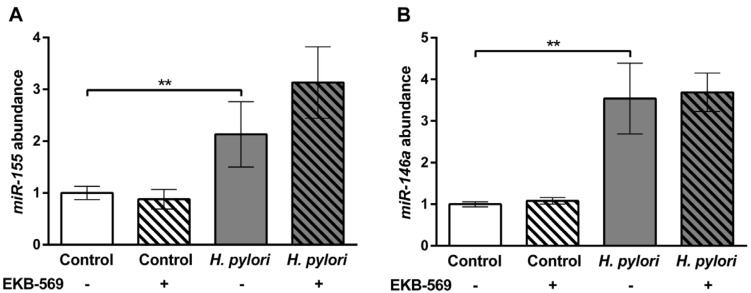
*H. pylori* infection increases miR-155 and miR-146a abundance in the gastric mucosa which is not modified by EGFR inhibitor treatment. Mongolian gerbils were infected with *H. pylori* SS1 strain for 38 weeks. Real-time PCR was used to quantify the gastric mucosal transcript abundance of (**A**) *miR155* and (**B**) *miR146* in *H. pylori* infected and control gerbils. Grey histograms, *H. pylori* infected gerbils; white histograms uninfected control gerbils; Hatched bars infected and control groups treated with EKB-569 diet, unhatched bars infected and control groups on control diet. Group size n = 7–10 gerbils. Results are expressed as mean ± SEM. ** *p* < 0.01 Mann-Whitney Test.

As *Ifn-γ* transcripts in the gastric mucosa of *H. pylori-*infected gerbils have previously been shown to correlate with the grade of chronic inflammation [10], *Ifn-γ* transcripts were compared in *H. pylori-*infected EKB-569 treated and untreated gerbils. Transcripts were quantified using the 2^−ΔΔCt^ method [32] using *Gapdh* as a reference gene. No significant difference was evident in *Ifn-γ* transcript abundance in *H. pylori-*infected gerbils on EKB-569 or control diet (data not shown) concurring with their similar chronic inflammatory scores (Figure 1). The miRNAs *miR-155* and *miR-146a* are increased in human gastric mucosa in *H. pylori* infection [33,34]. miRNAs were quantified using the 2^−ΔΔCt^ method [32] using U6 snRNA as a reference gene. Quantitative PCR showed transcript abundances of miR-146a and miR-155 were also significantly increased (*p* < 0.01) in *H. pylori* SS1-infected gerbils compared to uninfected controls (Figure 5A,B). There was no significant difference in gastric *miR-146a* and *miR-155* transcript abundance in infected gerbils on EKB-569 or control diet.

**Figure 6:**
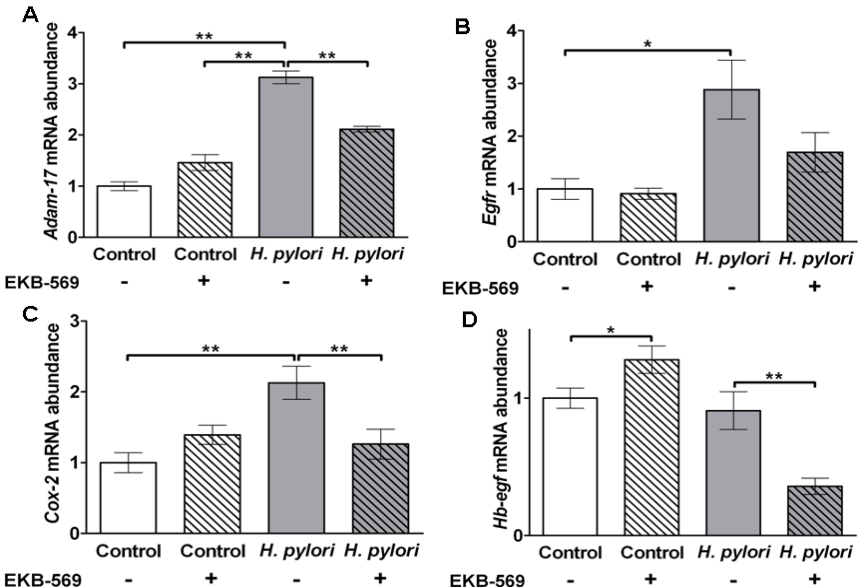
EGFR inhibitor treatment decreases *H. pylori* induced *Adam17*, *Egfr* and *Cox2* transcript abundance in gastric mucosa. Gerbils were infected with *H. pylori* SS1 strain for 38 weeks. Real-time PCR was used to quantify the gastric mucosal transcript abundance of (**A**) *Adam17*; (**B**) *Egfr*; (**C**) *Cox-2*; (**D**) *Hb-egf* in *H. pylori* infected gerbils and controls. Grey histograms *H. pylori* infected gerbils, white histograms uninfected control gerbils; Hatched bars infected and control groups treated with EKB-569 diet, unhatched bars infected and control groups on control diet. Group size n = 7–10 gerbils. Results are expressed as mean ± SEM. * *p* < 0.05; ** *p* < 0.01 Mann-Whitney Test.

Analysis of *Adam-17*, *Egfr* and *Cox-2* in the gastric mucosa of uninfected control gerbils showed that EKB-569 had no significant effect on transcript abundance of these genes (Figure 6A–C). In contrast *H. pylori* infection resulted in significant increases in gastric mucosal *Adam17* (*p* < 0.01), *Egfr* (*p* < 0.05) and *Cox-2* (*p* < 0.01) transcript abundance in gerbils on control diet (Figure 6A–C). EKB-569 treatment of *H. pylori-*infected gerbils significantly reduced the abundance of *Adam17* (*p* < 0.01), *Cox-2* (*p* < 0.01) and decreased *Egfr* gastric mucosal transcripts (Figure 5A–C). *HB-EGF* expression is upregulated in cultured human gastric epithelial cells by *H. pylori* [14,35] and HB-EGF is also present in parietal cells [36]. EKB-569 treatment of uninfected gerbils resulted in increased *Hb-egf* transcripts (*p* < 0.05) (Figure 6D). No increase in *Hb-egf* transcripts was observed in *H. pylori-*infected gerbils on control diet relative to uninfected controls on control diet. However in *H. pylori-*infected gerbils a significant decrease (*p* < 0.01) was evident in animals treated with EKB-569 (Figure 6D).

## 3. Discussion

*H. pylori* can directly transactivate the EGFR on epithelial cells [14,15,16] and peptides such as gastrin [37], chemokines [38] and molecules such as PGE_2_ [39] which are upregulated by *H. pylori* will also, via their specific G coupled protein receptors, induce EGFR transactivation. Thus, *in vivo*, multiple pathways induced by *H. pylori* may contribute to EGFR transactivation via the TMPS cascade, which involves extracellular cleavage of membrane bound EGFR ligands such as proHB-EGF and amphiregulin by ADAM metalloproteases [4].

EKB-569 is a potent selective irreversible inhibitor of EGFR tyrosine kinase activity both *in vitro* and *in vivo* in rodents at clinically relevant doses [30,40]. EKB-569 also has reduced activity against ErbB-2 (HER2/neu) [30] which is also activated by *H. pylori* [41]. EKB-569 administration in dietary formulation blocks intestinal polyp formation in APC^Min/+^ mice [30] and combination therapy with cyclooxygenase inhibitors results in synergistic prevention of polyp development [30]. This latter study only investigated polyp formation and did not evaluate any effects of EKB-569 on epithelial kinetics and intestinal gene expression. 

In the present study EKB-569 significantly reduced *H. pylori* phosphorylation of ERK1/2 in gastric epithelial cells both *in vitro* and *in vivo*. In addition, nuclear phospho-Erk was mainly observed in *H. pylori* infected gerbils on control diet. Previous studies indicate nuclear accumulation of Erk2 depends on phosphorylation state and dimerization [42]. Phospho-Erk positive mononulcear cells in the lamina propria were present in infected gerbils on both control diet and EKB-569 diet. Although EGFR is strongly expressed on the basal lateral membranes of gastrointestinal epithelial cells [43], EGFR is also present in myeloid cells [44] and fibroblasts [45] which are increased in *H. pylori* infection [46]. The lack of specific reagents for gerbils precluded detailed analysis of the lamina propria specific mononuclear cell populations. The potential effects of EKB-569 on the function of lamina propria mononuclear cell populations should be considered in future studies given the importance of epithelial-stromal interactions in disease pathogenesis [46]. 

In the present study *in vivo* EKB-569 treatment of gerbils during weeks 6 to 38 post-infection had no effect on density of *H. pylori* colonisation or gastric inflammation determined histologically and by assessment of gastric *Ifnγ*, *miR-146a* and *miR-155* transcripts, respectively. Gastric *miR-146a* and *miR-155* transcripts, which were significantly increased in *H. pylori* infected gerbils on both EKB-569 and control diets, negatively regulate the inflammatory response to *H. pylori* [33,34]. In contrast, in *H. pylori* infected gerbils, EKB-569 treatment was associated with significantly reduced corpus atrophy (the pre-neoplastic lesion end point), mucous metaplasia and glandular submucosal herniations and a marked reduction in antral and corpus epithelial to apoptosis ratios. Consistent with previous studies with *H. pylori* SS1 strain [10], no gerbils in this study had dysplasia or cancer. 

EKB-569 treatment of infected gerbils significantly increased gastric epithelial apoptosis relative to infected gerbils on control diet at 38 weeks post-infection. Enhanced gastric epithelial cell apoptosis and the reduced proliferation to apoptosis ratio may account for the significant reduction in submucosal herniations, the latter being a proliferative response considered to be a first-step in the development of adenocarcinoma in this model. Whether enhanced apoptosis contributes to the significant reduction in corpus atrophy and a diminished requirement for mucous metaplasia requires further investigation. Earlier studies on murine airway epithelium have shown EKB-569 interrupts viral induced anti-apoptotic pathways and partially inhibits goblet cell metaplasia [47]. Previous studies have shown a significant reduction in gastric epithelial cell apoptosis in gerbils at 38 weeks post-infection with *H. pylori* SS1 strain relative to 12 weeks post-infection [10]. The *H. pylori* CagA protein can inhibit vacuolating cytotoxin induced apoptosis in epithelial cell lines [48]. However as the SS1 strain does not have a functional *cag* PAI [49], epithelial CagA translocation is unlikely to be involved in the decline in epithelial apoptosis in untreated infected gerbils. 

The increased gastric epithelial apoptosis in EKB-569 treated infected gerbils was associated with a significant decrease in *Cox-2*, *Adam17* and *Egfr* gastric transcripts. Gastric cyclooxygenase-2 is increased in *H. pylori* infection [50] and elevated levels of PGE_2_ will transactivate the EGFR [39] decreasing apoptosis. Reduction of *Cox-2* gastric transcripts in EKB-569 treated gerbils is in line with previous studies showing that upregulation of COX-2 protein in human [51] and murine [52] gastric epithelial cell lines by *H. pylori* is dependent on EGFR transactivation. Furthermore, pharmacological inhibition of Cox-2 upregulation in *H. pylori* stimulated murine gastric epithelial cells increases apoptosis [52]. Clinically in *H. pylori* infected patients with gastric ulcers the use of non-steroidal anti-inflammatory drugs reduces the risk of gastric cancer [53]. The synergistic effects of cycloxygenase inhibitors and EKB-569 on polyp formation in APC^Min/+^ mice [30] may similarly result from increased apoptosis. 

Surprisingly no significant increase in gastric *Hb-egf* transcripts was observed in untreated gerbils with *H. pylori* infection relative to uninfected controls, although a significant reduction of *Hb-egf* transcript abundance was evident in infected gerbils with EKB-569 treatment. Although antral mucosa was used to avoid *Hb-egf* positive parietal cells [36], possible parietal cells in the transition zone in two control gerbils with high *Hb-egf* transcripts may have precluded the expected increase in *Hb-egf* transcripts with *H. pylori* infection. *In vitro* studies with human MKN-28 gastric cancer cells have shown *H. pylori* induced upregulation of *COX-2* and *HB-EGF*, but not *ADAM17,* is reduced by EKB-569 treatment [54]. EGF stimulated upregulation of *COX-2* and *HB-EGF*, but not *ADAM17*, in human MKN-28 gastric epithelial cells are also downregulated by EKB-569 treatment [54]. 

The membrane metalloprotease ADAM17 has a critical role in cleaving proHB-EGF on gastric epithelial cells following *H. pylori* stimulation resulting in EGFR transactivation by mature HB-EGF [14,18]. *In vitro* studies indicate that ADAM17 is required for the anti-apoptotic effect of *H. pylori* [18]. The significant decrease in *Egfr* transcripts in the gastric mucosa of infected gerbils with EKB-569 treatment probably reflects an overall reduction in gastric epithelial cells. These *in vivo* observations in gerbils concur with previous studies in an immortalised gastric epithelial cell line in which EGFR knockdown by siRNA enhanced *H. pylori* stimulated apoptosis [18]. 

In gastric cancer the expression of *ADAM10*, *ADAM15*, *ADAM17* and *ADAM 20* transcripts is markedly increased [19]. In addition, increases in EGF-related peptides HB-EGF and amphiregulin are evident in patients with *H. pylori* infection and/or gastric cancer [20,22]. Upregulation of these key components for EGFR transactivation by *H. pylori* could promote autocrine signaling loops promoting epithelial hyperproliferation and block apoptosis. 

Although no significant reduction in gastric epithelial proliferation was identified with EKB-569 treatment in infected gerbils, *in vivo* rodent studies with other carcinogens such as asbestos, which also transactivates the EGFR [55], indicate bronchial hyperproliferative responses are dependent on EGFR activation [56]. Asbestos induced inflammation alone in transgenic mice with mutant EGFR was not associated with epithelial hyperproliferative responses nor proto-oncogene activation [56]. In *H. pylori* SS1-infected gerbils gastric epithelial proliferation, but not apoptosis, correlates with active and chronic inflammatory scores [10]. The lack of reduction in epithelial cell proliferation with EKB-569 treatment in the present study is thus consistent with absence of changes in gastric inflammation in this model. Studies in immune deficient transgenic mice also indicate the important role of gastric inflammation in development of epithelial hyperproliferative responses to gastric *Helicobacter* infection [57]. The lack of effect of EKB-569 on antral epithelial proliferation in infected gerbils suggests multiple pathways may contribute to the epithelial hyperproliferative response. 

## 4. Experimental

### 4.1. H. pylori Culture

*H. pylori* SS1 strain was grown on blood agar base with 7% (v/v) horse blood under microaerobic conditions at 37 °C. For *in vitro* experiments two day cultures were harvested into antibiotic-free RPMI 1640 medium and suspended at a concentration of 2.5 × 10^7^ organisms/mL. For *in vivo* experiments two day SS1 cultures were harvested into sterile tryptose soya broth and used immediately for inoculation into gerbils. 

### 4.2. *In Vitro* Bacterial-Epithelial Co-Culture

A-431 (ATCC CRL-1555) (American Type Culture Collection, Manassas, VA, USA) human epidermoid carcinoma cells, which express high levels of EFGR, were used. To examine the effects of EKB-569 on *H. pylori* SS1-stimulated ERK1/2 activation over night serum starved A-431 in 96 well plates (Nunc, Rochester, NY, USA)were pre-incubated with EKB-569 at concentrations of 0, 0.01, 0.1, 0.5, 1.0 µM/well for 1hr prior to co-culture with *H. pylori* SS1 for 3h as previously described [16]. EKB-569 was kindly provided by L. Greenberger (Wyeth Research Chemical Sciences, Pearl River, NY, USA). Inhibition of *H. pylori-*stimulated ERK1/2 phosphorylation was quantified by “In Cell Western” assay using mouse mouse-monoclonal anti-pERK1/2 (Santa Cruz, Dallas, TX, USA) and rabbit-polyclonal anti-ERK1 (Santa Cruz) antibodies as previously described [16]. The 700 nm Relative Value (Rel_700_) was obtained after normalization with 800 nm values to control for cell density variation [16]. 

### 4.3. Infection of Mongolian Gerbils with H. pylori

Male Mongolian gerbils aged 6–8 weeks old (supplied by MGS/Sea, Seac Yoshimoto, Fukuoka, Japan and bred at the University of Leeds) were inoculated three times by oral gavage with *H. pylori* SS1 (>10^8^ CFU) over a 5 day period. Inoculated and control gerbils were maintained under a 12 h light/dark cycle, temperature 19–23 °C and humidity 45%–55%. After six weeks inoculated and control animals (n = 8–10) received *per os* 10 mgs/kg/day/gerbil EKB-569 in dietary formulation or control. Previous rodent studies [30] have demonstrated the efficacy of this dose in long term inhibition of EGFR activity. All experimental procedures were approved by the local ethics committee of Leeds University and the UK Home Office. 

### 4.4. Histological and Microbial Analysis of H. pylori Infection

Gerbils were sacrificed at 38 weeks post-inoculation. One hour prior to sacrifice inoculated gerbils and controls received an intra-peritoneal injection of bromodeoxyuridine (BrdU) (50 mg/kg). At sacrifice, gastric tissue was taken for microbial culture, histology and snap frozen in liquid nitrogen and stored at −–70 °C for subsequent RNA extraction. *H. pylori* culture from gastric mucosa was on selective plates as previously described [12]. 

Gastric pathology was graded on haematoxylin and eosin stained sections by a pathologist in a blinded fashion as previously described [10,12]. The entire field of four sections of gastric mucosa was evaluated histologically to give one score for antral and one score for corpus mucosa per gerbil. Gerbils were scored for active and chronic inflammation, atrophy, and mucous metaplasia. All parameters were graded on a scale of 0–3, with 0 being histologically normal, 1- mild, 2- moderate and 3- severe abnormality. In addition, the extent of herniations below the muscularis mucosa was scored on a scale 0–3, with a score of zero being no herniations, 1- mild, 2- moderate and 3 severe submucosal herniations. Density of *H. pylori* in antrum and corpus was assessed on Giemsa stained sections as previously described [10]. Gastric epithelial cell proliferation was assessed by BrdU immunohistochemistry as previously described [10,11,12]. Epithelial cell proliferation was expressed as a labelling index (LI%) ([percentage of stained cells/total cells per gastric gland] ×100). 

Apoptotic epithelial cells were identified immunohistochemically with a rabbit polyclonal antibody against active caspase 3 (Cell Signalling Technology, Danvers, MA, USA). Glandular epithelial apoptosis was expressed as an apoptotic index (AI%) ([percentage of stained cells /total cells per gastric gland] ×100) as previously described [10,11]. Quantitative assessments were undertaken on a Nikon E1000 microscope (Nikon Inc., Melville, NY, USA). To evaluate changes in epithelial cell proliferation and apoptosis, a score for epithelial cell kinetics in individual gerbils was determined by dividing the epithelial proliferation index (LI%) by the apoptotic index (AI%) [7].

Immunohistological analysis of phospho-p44/p42 Map Kinase (Erk 1/2) (Thr202/Tyr204) activation in the gastric epithelium was undertaken using a rabbit monoclonal antibody (4370) (Cell Signaling Technology) and EnVision+Dual System-HRP (DAB+) (Dako, Carpinteria, CA, USA). The number of phospho-Erk positive gastric epithelial cells per high-power field was determined as previously described [58].

### 4.5. Sequence Analysis of Mongolian Gerbil Genes

Primers specific for *Adam17*, *Egfr* and *Hb-Egf* transcripts were designed by cross species PCR after aligning respective murine, rat and human sequences and identifying regions of homology. PCR products were sequenced as previously described [27].

### 4.6. Quantitative Polymerase Chain Reaction (qPCR)

Gastric mucosal RNA was extracted in Trizol (Invitrogen, Paisley, UK), DNase treated (Applied Biosystems, Warrington, UK) and reverse-transcribed with SuperScript II Reverse Transcriptase (Invitrogen, Paisley, UK) and random hexamers (Bioline, London, UK). Real-time qPCR amplification was performed using gene-specific oligonucleotide primers for *Adam17*, *Cox-2*, *Egfr*, *Hb-egf*, *Ifn-γ*, and *Gapdh* (Table 2) and SYBR Green PCR Master Mix (Applied Biosystems). Quantitative PCR amplification consisted of an initial denaturing step at 95 °C for 10 min, 40 cycles of 95 °C for 15 s and 60 °C for 1min. Reactions were performed in a 7500 Real Time PCR System (Applied Biosystems) and quantified using 2^−ΔΔCt^ method [32] using *Gapdh* as a reference gene. 

**Table 2:** Primers used for quantitative real-time polymerase chain reaction (q-PCR).

Gene	Primers	Product size (bp)
***Adam17***	5'-AAAGGGAACCCTGTACCGTAGGG 5'-GCCAAAAACTTTCCGAAAGTGT	131
***Cox-2***	5'-AGTCTCTCAACGAATACCGCAAAC5'-ATGTCACTGTAGAGGGCTTTCAAC	117
***Egfr***	5'-GGGAAATGCTCTGTACGAAAACAC5'-AGCACCGGTCAGGATTTCCT	118
***Gapdh***	5'-CCTGTGACTTTAACAGCGACTCC5'-CCATGAGGTCCACCACCCT	102
***Hb-egf***	5'-TCGGAGAGGTCTGGCGG5'-TCCTGGACTTCCTGAGTGCG	118
***Ifn-γ***	5'-CCATGAACGCTACACACTGCATC5'-GAAGTAGAAAGAGACAATCTGG	230
**miRNAs**	**Forward Primers**	
***U6 snRNA***	5'-dTGGCCCCTGCGCAAGGATG	-
***miR-146a***	5'-dTGAGAACTGAATTCCATGGGTT	-
***miR-155***	5'-dTTAATGCTAATTGTGATAGGGGT	-

### 4.7. Quantitative PCR Analysis of miRNAs Expression in Gerbil Gastric Mucosa

miRNAs were reverse-transcribed from extracted gastric RNA using the miRNome microRNA Profilers QuantiMir™ kit (System Biosciences; Mountain View, CA, USA) following the manufacturer’s instructions. Quantitative PCR for *miR-146a* and *miR-155* was performed by real-time PCR, using U6 snRNA as reference gene using forward primers detailed in Table 2 and the 3' universal reverse primer (System Biosciences). Quantitative PCR was as above. miRNAs were quantified using the 2^−ΔΔCt^ method [32] using U6 snRNA as a reference gene. 

### 4.8. Statistics

Results are presented as means ± S.E.M. Comparisons between groups were made using either Mann-Whitney U test, paired *t* test, stratified Chi squared for trend or Fishers exact test. A *p* value of less than <0.05 was considered significant.

## 5. Conclusions

In conclusion, this study demonstrates that treatment of *H. pylori* infected gerbils with a selective EGFR inhibitor improves gastric corpus pathology and precancerous lesions and substantially decreases both the antral and corpus epithelial to apoptosis ratios to those of uninfected gerbils. A significant increase in apoptosis in infected gerbils treated with the EFGR inhibitor was associated with reduced mucosal transcripts of *Cox-2*, *Adam17* and *Egfr*. These studies indicate for the first time a pathogenic role of *H. pylori* EGFR transactivation *in vivo* during long term chronic *H. pylori* infection. Targeting pre-malignant proliferative signalling pathways in non-malignant conditions with tyrosine kinase inhibitors is considered to have therapeutic potential for some conditions [59]. Whether such an approach with EGFR inhibitors would have a chemopreventative role in subjects who are at high risk of developing gastric cancer, who have failed *H. pylori* eradication therapy, requires consideration. 

## Supplementary Files

Supplementary File 1 Supplementary Material (PDF, 162 KB)
