# Supplementary material for: Effects of EGFR Inhibitor on Helicobacter pylori Induced Gastric Epithelial Pathology in Vivo
Source: Pathogens. 2013 Oct 14;2(4):571–90. doi: 10.3390/pathogens2040571 (PMC4235704; doi:10.3390/pathogens2040571)
Supplement: Supplementary File 1 — Supplementary Material (PDF, 162 KB) [file pathogens-02-00571-s001.pdf]

Supplementary Material for

Effects of EGFR Inhibitor on *Helicobacter pylori* Induced Gastric Epithelial Pathology *in Vivo*

Figure S1. Sequence alignment of Mongolian gerbil *Adam17*, *Egfr* and *Hb-egf.* (**A**) Alignment of 213 bp portion *Adam17* cDNA sequence for the Mongolian gerbil, mouse, rat, Chinese hamster and human. Those nucleotides in other species which are similar to those in the Mongolian gerbil are typed in stars, while those different are typed in letters. The degree of nucleotide identity observed between Mongolian gerbil and other species were 95% for mouse (Accession No. 9945314), 95% for rat (Accession No. 9945329), 95% for Chinese hamster (Accession No. AF380348) and 94% for human (Accession No. 14722411); (**B**) Alignment of 216 bp portion *Egfr* cDNA sequence for the Mongolian gerbil, mouse, rat, Chinese hamster and human. Those nucleotides in other species which are similar to those in the Mongolian gerbil are typed in stars, while those different are typed in letters. The degree of nucleotide identity observed between Mongolian gerbil and other species was 92% for mouse (Accession No. AF275367), 91% for rat (Accession No. M37394), and 80% for human (Accession No. X00588); (**C**) Alignment of 200 bp portion (*Hb-egf*) cDNA sequence for the Mongolian gerbil, mouse, rat, Chinese hamster and human. Those nucleotides in other species which are similar to those in the Mongolian gerbil are typed in stars, while those different are typed in letters. The degree of nucleotide identity observed between Mongolian gerbil and other species was 93% for mouse (Accession No. 6754177), 93% for rat (Accession No. L05489), 90% for Chinese hamster (Accession No. AF069753) and 79% for human (Accession No. M60278). Nucleotides identical to those in the Mongolian gerbil are indicated by *; Numbers in the right hand column are the nucleotide position assigned in each of the respective database entries. Primer sites for qPCR in bold.

A. *Adam17* sequence

CGTACGTCGATGCAGAGCAAAAGAACTTATTTTTGAGG**AAAGGGAAGCCC** 50 Gerbil
****************************G********************A 1887 Mouse
*T** 2128 Rat
C***T*** 1872 C.hamster
*C**T**************************************A****** 2051 Human

TGTACAGTAGGGTTTTGTGATATGAATGGAAAATGTGAAAAACGAGTACA 100 Gerbil
*****************C**C********C********G*********** 1937 Mouse
********************C********C********G*****G***** 2178 Rat
********************C*****C**C*****************T** 1922 C.hamster
***********A********C********C********G*********** 2101 Human

GGATGTAATTGAGCGATTTTGGGATTTCATTGACCAGCTGAGCATCA**ACA** 150 Gerbil
***C** 1987 Mouse
C********G********************************** 2223 Rat
** 1972 C.hamster
************A***********************************T* 2151 Human

CTTTCGGAAAGTTTTTGGCAGACAACATCGTTGGGTCTGTTCTGGTTTTC 200 Gerbil
****T**G******C*******T*************************** 2037 Mouse
****T**G******C*********************************** 2273 Rat
****T**G***********************A*****C**C********* 2022 C.hamster
****T***********A***********************C********* 2201 Human

TCCTTGATATTTT 213 Gerbil
************* 2050 Mouse
************* 2286 Rat
************* 2035 C.hamster
************* 2014 Human

B. *Egfr* sequence

TGGCTGGCTATGTGCTCATTGCCCTCAACACTGTGGAGAGGATCCCTCTGGAGAACCTGC 60 Gerbil
****C********C*****************C********A******T************ 536 Mouse
*************T***********G*****C********A******T************ 466 Rat
*******T*****C*****************A******C*A**T***T****A******* 499 Human

AAATCATCAG**GGGAAATGCTCTGTACGAAAACAC**CTATGCCTTAGCCGTCCTGTCCAACT 120 Gerbil
*G********************T**T*********************A************ 596 Mouse
*G********************C**************C********************** 526 Rat
*G********A******ATGTAC********TT*************A***T*A**T**** 559 Human

ACGGGGCAAACAGAACTGGGCTCAGGGAACTGCCTATGCGGAACTTAC**AGGAAATCCTGA** 180 Gerbil
*T***A****************T***********C************************* 656 Mouse
*T**AA*C****A*********T***********C********************T**** 586 Rat
*T*AT*****T*A***C**A**G*A***G*****C***A*A**T***************C 619 Human

CCGGTGCTGTGCGGTTCAGCAACAACCCCATCCTCT 216 Gerbil
TT***********A********************** 692 Mouse
T************A**T******************* 622 Rat
AT**C**C**************************** 647 Human

Figure S1. *Cont.*

C. *Hb-egf* sequence

GTGTTGTCTGCGTTGGTGACCGGTGAGAGTCTGGAGCGGCT**TCGGAGAGG** **50** Gerbil
** 356 Mouse
** 126 Rat
********************************A**A********C***** 102 C.hamster
TC*CG*CAC****************C******************** 356 Human

TCTGGCGGCAGCAACCAGCAACCCTGACCCTCCTACTGGATCCACACACC 100 Gerbil
*********************************C************A*** 406 Mouse
*********************************C******A*****A*** 176 Rat
*****A**********************T***********C*****A*** 152 C.hamster
G**A**T**T*G************G********C****T******GG*** 406 Human

AGCTGTTACCCACGGGAGGCGAT**CGCACTCAGGAAGTCCAGGA**CTTGGAT 150 Gerbil
*****C*************T*****TG******GG**************A 456 Mouse
*****C************CT******G*******G**************A 226 Rat
*****C************AT*G***TGG*********************A 202 C.hamster
*****C*****CTA*******GC**GGAC*G*A******GT******C*A 456 Human

AAGACAGACCTGGACATTTTCAGAGTTGCTTTCTCCTCCAAGCCACAAG 200 Gerbil
GG******T***A**C******A************************** 506 Mouse
GG***C**T******C******A************************** 276 Rat
G*******T*C****C********************************* 252 C.hamster
G**G****T******C****G*****CA****A**************** 406 Human
